# Assessment of central vein sign and paramagnetic rim lesions in pediatric multiple sclerosis

**DOI:** 10.1002/acn3.52208

**Published:** 2024-09-18

**Authors:** Monica Margoni, Paolo Preziosa, Elisabetta Pagani, Loredana Storelli, Mor Gueye, Lucia Moiola, Massimo Filippi, Maria A. Rocca

**Affiliations:** ^1^ Neuroimaging Research Unit, Division of Neuroscience IRCCS San Raffaele Scientific Institute Milan Italy; ^2^ Neurology Unit IRCCS San Raffaele Scientific Institute Milan Italy; ^3^ Neurorehabilitation Unit IRCCS San Raffaele Scientific Institute Milan Italy; ^4^ Vita‐Salute San Raffaele University Milan Italy; ^5^ Neurophysiology Service IRCCS San Raffaele Scientific Institute Milan Italy

## Abstract

The evaluation of white matter lesions (WMLs) showing the central vein sign (CVS) and paramagnetic rim lesions (PRLs) has been suggested to enhance the diagnostic work‐up of adult multiple sclerosis (MS). We aimed to evaluate the fulfillment of different CVS criteria and the added value of PRLs in 22 pediatric MS patients. Eleven patients (50%) fulfilled the 40%‐rule threshold. Nineteen (86%) patients had ≥3 CVS+ WMLs or ≥1 PRL, whereas 17 (77%) had ≥6 CVS+ WMLs or ≥1 PRL. A simplified CVS‐based approach, with the combined evaluation of ≥1 PRL in patients with ≥6 CVS+ WMLs, may improve MS diagnosis in pediatric patients.

## Introduction

Identifying specific MRI features for multiple sclerosis (MS) is crucial for early diagnosis and minimizing the risk of misdiagnosis.

The central vein sign (CVS) is a central linear hypointensity identifiable within some white matter lesions (WMLs) on susceptibility‐based MRI sequence, corresponding to the venules around which the lesion developed.^1^ Paramagnetic rim lesions (PRL) are a subgroup of WMLs also observable with this sequence, characterized by a hypointense rim related to the presence of iron‐laden activated microglia/macrophages associated with ongoing demyelination and axonal loss.^2^ The CVS+ WMLs and PRLs are more frequent in MS compared to other conditions, such as migraine, neuromyelitis optica spectrum disorders, myelin oligodendrocyte glycoprotein antibody‐associated disease (MOGAD), inflammatory CNS disorders, and cerebral small‐vessel disease.^3^, ^4^, ^5^

Various approaches have been proposed to define the fulfillment of CVS criteria in adult MS patients, including threshold‐based methods (a minimum proportion of CVS+ WMLs, such as 35%, 40%, and 50%)^6^ or simplified rules based on the presence of at least three or six CVS+ WMLs (i.e., three‐ or six‐lesion rules).^5^, ^6^, ^7^, ^8^, ^9^ These simplified approaches may be more practical in clinical setting, especially for patients with numerous WMLs. Recently, the presence of at least one PRL, especially if combined with CVS assessment, allowed to distinguish MS from mimickers and aged controls^10^ with high accuracy and to confirm MS based on dissemination in time and space criteria.^11^

The CVS+ WMLs and PRLs have been evaluated in limited cohorts of pediatric MS patients (range: 10–26 subjects).^2^, ^12^, ^13^ The proportion of CVS+ WMLs ranged from 51% to 63%, whereas the threshold of 40% of CVS+ WMLs was met in 70%–100% of pediatric MS patients.^12^, ^14^ Conversely, PRLs were described in 69–77% of pediatric MS patients.^2^, ^12^ Notably, the CVS‐positive rate and PRLs have been identified as highly specific markers of pediatric MS, distinguishing it from MOGAD.^12^ No study has evaluated the fulfillment of the three‐ or six‐lesion rules in these patients and their combination with PRLs.

Given this background, we explored the fulfillment of different CVS criteria, not only specific thresholds, but also three‐ and six‐lesion rules, and the added value of PRLs in pediatric MS patients.

## Methods

### Standard protocol approvals, registrations, and patient consents

Approval was received from the local ethical standards committee on human experimentation (protocol ID: 25/2007), and written informed consent was obtained from all participants and their parents prior to study enrollment.

### Participants

This is a cross‐sectional analysis of a prospective cohort of 22 pediatric MS patients. To be included, they had to have a diagnosis of MS according to the 2017 revised McDonald criteria and be relapse‐ and steroid‐free for at least 1 month prior to clinical and MRI assessment. Exclusion criteria was a history of other neurological/psychiatric disorders in addition to MS. Whenever needed, appropriate testing was performed to exclude MOGAD.

On the day of MRI, patients underwent a complete neurologic evaluation, with rating of the Expanded Disability Status Scale (EDSS) score and recording of ongoing disease‐modifying treatments (DMTs).

### MRI acquisition

Using a 3.0‐T Philips Achieva dStream MR scanner (Philips Medical System), the following brain sequences were acquired from all subjects: (i) three‐dimensional (3D) T2 fluid‐attenuated inversion recovery (FLAIR), field of view (FOV) = 256 × 256 mm, voxel size = 1 × 1 × 1 mm, 192 slices, matrix = 256 × 256, repetition time (TR) = 4800 ms, echo time (TE) = 268 ms, inversion time (TI) = 1650 ms, echo train length (ETL) = 167, TA = 6.15 min; (ii) 3D T1‐weighted turbo field echo, FOV = 256 × 256, voxel size = 1 × 1 × 1 mm, 204 slices, matrix = 256 × 256, TR = 7 ms, TE = 3.2 ms, TI = 1000 ms, flip angle = 8°, acquisition time (TA) = 8.53 min; and (iii) 3D T2*, FOV =230 × 230, pixel size = 0.60 × 0.60 mm, 135 slices, 2 mm‐thick reconstructed to 1 mm, matrix = 384 × 382, TR = 39 ms, TEs = 5.5:6:35.5 ms, flip angle = 17°, TA = 6 min; both magnitude and raw phase images for each echo were saved.

### MRI analysis

WMLs were identified and their mask was obtained for each patient using a fully automated and validated approach using the 3D FLAIR and 3D T1‐weighted as input images, after a careful visual check of the results provided by the automatic segmentation.

The 3D FLAIR image was registered onto the T2* space through rigid transformations using FLIRT embedded in the FSL; the same transformation was applied to WML masks. Then the transformed FLAIR was multiplied with the T2* image with the longest echo time from the multiecho sequence to obtain the FLAIR* contrast.^1^

**Figure 1:**
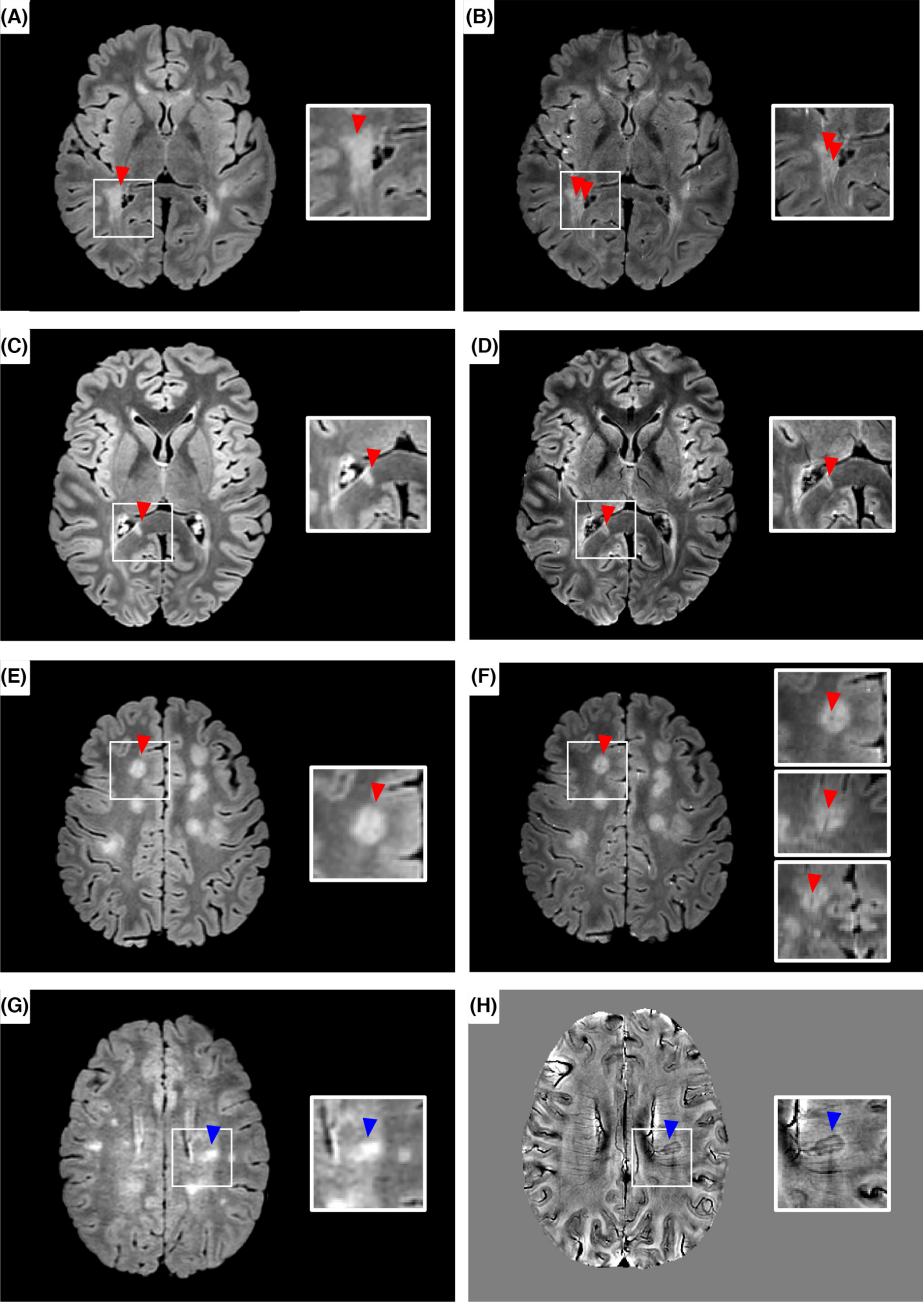
White matter lesions with central vein sign and paramagnetic rim in patients with pediatric multiple sclerosis. Axial fluid‐attenuated inversion recovery (FLAIR) sequence showing a confluent lesion (A) with multiple veins within its context on FLAIR* (B). Axial (C,E) FLAIR sequences showing brain T2‐hyperitense white matter lesions that, on FLAIR* (D,F) demonstrate a central vein sign (red triangles). In (F) the central vein sign is visible in the three plains (axial, sagittal, and coronal). Axial FLAIR sequence (G) showing a brain T2‐hyperitense white matter lesion that, on phase image (H), demonstrates a paramagnetic rim (blue triangle). See text for further details.

WMLs were excluded from the CVS assessment if they were <3 mm, visible on a single plane, merged with other lesions, or presented multiple distinct veins in their context (Fig. 1). The CVS was manually assessed on FLAIR* imaging using T2‐hyperintense WM lesion masks as support (Fig. 1).

PRLs were assessed on phase images after unwrapping and removal of global spatial changes, as previously described.^2^ According to the most recent consensus, PRLs were defined as lesions characterized by a T2‐hyperintense core, surrounded by a paramagnetic rim on the susceptibility sequence that encompasses at least two‐thirds of the lesion's perimeter, and showing no gadolinium enhancement on a post‐contrast T1‐weighted image (previously obtained on a recent clinical scanner).^15^

All analyses were performed by two trained raters (P.P. and M.M., neurologists with 15 and 10 years of experience). Both raters were trained by M.A.R. (neurologist with 25 years of experience). Intra‐observer and inter‐observer reproducibility were assessed by evaluating the CVS and PRL of eight randomly selected pediatric MS patients twice by both raters, with a minimum interval of 15 days between assessments. Intraclass correlation coefficients (ICCs) were derived from a two‐way random effects model.

For each patient, the number of total WMLs, CVS+ WMLs and PRLs and excluded WMLs were automatically estimated using an in‐house implemented method with Matlab v2012. For the CVS, the percentage of perivenular lesions across all eligible brain lesions was determined in each participant. Accordingly, different criteria were evaluated, including a minimum threshold of percentage of CVS+ WMLs (i.e., 35%, 40%, and 50%),^1^, ^4^, ^5^ or simplified approaches based on the presence of three (three‐lesion rule)^9^ or six (six‐lesion rule)^16^ CVS+ WMLs. Lesions were also categorized as either juxtacortical (adjacent to the cortex), periventricular (abutting to the ventricles), infratentorial or deep WMLs. Combined fulfillments of CVS+ criteria and presence of at least one PRL were also evaluated.

## Results

Pediatric MS patients had a median age of 16.4 years (interquartile range [IQR] = 13.0; 17.4 years), a median disease duration of 0.7 years (IQR = 0.4; 1.3 years) and a median EDSS of 1.0 (IQR = 1.0; 2.0). All patients had cerebrospinal fluid‐specific oligoclonal bands and 16 patients were under DMTs (Table 1).

**Table 1:** Clinical and magnetic resonance imaging characteristics of participants with pediatric multiple sclerosis.

Characteristic	Value
Patients, No.	22
Age, median (IQR), years	16.4 (13.0; 17.4)
Girls (%)	19 (86)
Disease duration, median (IQR), years	0.7 (0.4; 1.3)
Patients with oligoclonal bands (% of cases with CSF)	22 (100)
EDSS, median (IQR)	1.0 (1.0; 2.0)
Patients in DMTs (%)	16 (73)
Total number of brain WMLs, No.	1564
Total number of brain WMLs per patient, median (IQR)	43 (16; 85)
Total number of excluded brain WMLs (confluent WMLs or with multiple veins), No.	250
Total number of excluded brain WMLs (confluent WMLs or with multiple veins) per patient, median (IQR)	6.5 (2; 19)
Total number of evaluable brain WMLs, No.	1225
Total number of evaluable brain WMLs per patient, median (IQR)	30 (13; 71)
Total brain CVS+ WMLs, No.	495
Total brain CVS+ WMLs per patient, median (IQR)	12 (4; 23)
Number of periventricular CVS+ WMLs per patient, median (IQR)	4 (0; 6)
Number of juxtacortical CVS+ WMLs per patient, median (IQR)	4 (0; 6)
Number of infratentorial CVS+ WMLs per patient, median (IQR)	0 (0; 2)
Number of deep CVS+ WMLs per patient, median (IQR)	4 (1; 10)
Total brain PRL+ WMLs, No.	35
Total brain PRL+ WMLs per patient, median (IQR)	1 (0; 2)
Number of patients with	
≥1 brain CVS+ WML (%)	21 (95)
≥3 brain CVS+ WMLs (%)	19 (86)
≥6 brain CVS+ WMLs (%)	15 (68)
Proportion of CVS+ WMLs per patient (%), median (IQR)	39 (33; 48)
Number of patients fulfilling CVS+ threshold	
≥35% (%)	14 (64)
≥40% (%)	11 (50)
≥50% (%)	5 (23)
Number of patients with	
≥1 brain PRL (%)	14 (64)
≥1 brain CVS+ WML or ≥1 PRL (%)	21 (95)
≥3 brain CVS+ WML or ≥1 PRL (%)	19 (86)
≥6 brain CVS+ WML or ≥1 PRL (%)	17 (77)

CSF, cerebrospinal fluid; CVS, central vein sign; DMT, disease‐modifying treatment; EDSS, Expanded Disability Status Scale; IQR, interquartile range; MRI, magnetic resonance imaging; PRL, paramagnetic rim lesions; WMLs, white matter lesions.

Manual CVS+ WMLs and PRLs identification showed a good reproducibility: the intra‐observer reliability was 0.93 (95% confidence interval [CI] = 0.86; 0.97) and the inter‐observer reliability was 0.87 (95% CI = 0.66; 0.94) for CVS+ WMLs; whereas, the intra‐observer reliability was 0.81 (95% CI = 0.13; 0.98) and the inter‐observer reliability was 0.75 (95% CI = 0.17; 0.96) for PRLs.

Of 1564 WMLs, 1225 (78%) were assessed for CVS and PRLs; 339 WMLs were excluded from the analysis. Two hundred and fifty confluent WMLs or with multiple veins within their context were excluded from the analysis. Four hundred and ninety‐five CVS+ WMLs (see Table 1 for their topographical distribution) and 35 PRLs were identified (Fig. 1).

The median numbers of CVS+ WMLs and PRLs per patient were 12 (interquartile range [IQR] = 4; 23) and 1 (IQR = 0; 2). Nineteen (86%) pediatric MS patients had ≥3 CVS+ WMLs and 15 (68%) patients had ≥6 CVS+ WMLs. The median proportion of CVS+ WMLs per patient was 39% (IQR = 33; 48). Eleven patients (50%) fulfilled the 40%‐rule threshold.

Fourteen (64%) pediatric MS patients had ≥1 PRL. Nineteen (86%) pediatric patients had ≥3 CVS+ WML or ≥1 PRL, whereas 17 (77%) had ≥6 CVS+ WM or ≥1 PRL.

## Discussion

In line with previous studies,^2^, ^12^, ^13^, ^14^ we detected a high number of CVS+ WMLs and PRLs in our pediatric MS patients, supporting the relevance of these lesions as clinically feasible and specific MRI markers for MS. Our results align with the evidence indicating perivenous demyelination and chronic inflammation as distinctive pathological features of MS, being found early in the disease continuum, including adult individuals with radiologically isolated syndrome^17^, ^18^ and patients with clinically isolated syndrome (CIS).^5^, ^11^ Although the proportion of CVS+ lesions per patient in pediatric MS patients was lower compared to that previously described in CIS patients (39% vs. 60%), we observed a higher percentage of patients fulfilling the three‐lesion rule (86% vs. 61.9%).^5^

Evaluating pediatric MS patients with short disease duration allowed us exploring the relevance of CVS and PRLs assessment in close proximity to clinical onset. This mirrors a scenario where specific MRI markers are needed for MS diagnostic work‐up in the clinical setting.

Our study showed that a higher percentage of patients met the criteria for the three‐ and six‐lesion rule compared to a proportion‐based CVS threshold. In pediatric MS, meeting the CVS+ criteria with a minimum of three or six CVS+ WMLs may facilitate diagnosis more effectively than relying on a proportion‐based CVS threshold. Indeed, compared to adults, pediatric MS patients may exhibit a higher number of WMLs, often with a confluent pattern,^19^, ^20^ limiting a direct translation of findings from adults to this patient population. Therefore, these factors make the assessment of CVS for each single lesion challenging, even for experienced raters, hindering an accurate assessment of the percentage of lesions with CVS. Although our findings need to be confirmed in a larger cohort of pediatric MS patients, our study suggests that the combined evaluation of PRLs could be helpful for MS diagnosis in those patients having at least six CVS+ WMLs.

This study has some limitations. First, we evaluated a relatively small cohort of patients; future studies investigating larger cohorts of patients are needed to better explore the diagnostic value of these lesions in this population. Second, the study was designed to evaluate the sensitivity of established CVS‐based criteria in combination with PRLs in pediatric MS patients. Although it lacks a non‐MS cohort to demonstrate the specificity of these lesions, a previous study already showed their discriminative power in differentiating pediatric MS from MOGAD.^12^ Third, our sequence does not achieve the 0.5‐mm isotropic resolution of the single‐echo sequences; this may have limited the identification of the CVS for venules oriented in the cranio‐caudal direction. Finally, we evaluated CVS+ WMLs and PRLs with a cross‐sectional approach. Future longitudinal studies are needed to explore dynamic changes of these lesions and their associations with measures of disease activity and progression.

In conclusion, a simplified CVS‐based approach, with the combined evaluation of at least one PRL in patients with at least six CVS+ WMLs, may be a relevant, feasible, and time saving additional diagnostic criterion for pediatric MS to be applied in the clinical setting.

## Funding Information

None.

## Conflict of Interest

The authors have no conflict of interest to declare.

## Author Contributions

M.M., P.P., M.F., and M.A.R. contributed to the conception and design of the study. M.M., P.P., E.P., L.S., M.G., L.M., M.F., and M.A.R. contributed to the acquisition and analysis of data, drafting the text and preparing the figures, and approved the final draft of the manuscript.

## Data Availability

The dataset used and analyzed during the current study are available from the corresponding author on reasonable request.
